# Deep learning approach to estimate foot pressure distribution in walking with application for a cost-effective insole system

**DOI:** 10.1186/s12984-022-00987-8

**Published:** 2022-01-16

**Authors:** Frederick Mun, Ahnryul Choi

**Affiliations:** 1 College of Medicine, The Pennsylvania State University, Hershey, USA; 2 Department of Biomedical Engineering, College of Medical Convergence, Catholic Kwandong University, 24, Beomil-ro 579, Gangneung, Gangwon 25601 Republic of Korea

**Keywords:** Gait, Foot pressure distribution, Deep learning, Long short-term memory, Cost-effective insole system

## Abstract

**Background:**

Foot pressure distribution can be used as a quantitative parameter for evaluating anatomical deformity of the foot and for diagnosing and treating pathological gait, falling, and pressure sores in diabetes. The objective of this study was to propose a deep learning model that could predict pressure distribution of the whole foot based on information obtained from a small number of pressure sensors in an insole.

**Methods:**

Twenty young and twenty older adults walked a straight pathway at a preferred speed with a Pedar-X system in anti-skid socks. A long short-term memory (LSTM) model was used to predict foot pressure distribution. Pressure values of nine major sensors and the remaining 90 sensors in a Pedar-X system were used as input and output for the model, respectively. The performance of the proposed LSTM structure was compared with that of a traditionally used adaptive neuro-fuzzy interference system (ANFIS). A low-cost insole system consisting of a small number of pressure sensors was fabricated. A gait experiment was additionally performed with five young and five older adults, excluding subjects who were used to construct models. The Pedar-X system placed parallelly on top of the insole prototype developed in this study was in anti-skid socks. Sensor values from a low-cost insole prototype were used as input of the LSTM model. The accuracy of the model was evaluated by applying a leave-one-out cross-validation.

**Results:**

Correlation coefficient and relative root mean square error (RMSE) of the LSTM model were 0.98 (0.92 ~ 0.99) and 7.9 ± 2.3%, respectively, higher than those of the ANFIS model. Additionally, the usefulness of the proposed LSTM model for fabricating a low-cost insole prototype with a small number of sensors was confirmed, showing a correlation coefficient of 0.63 to 0.97 and a relative RMSE of 12.7 ± 7.4%.

**Conclusions:**

This model can be used as an algorithm to develop a low-cost portable smart insole system to monitor age-related physiological and anatomical alterations in foot. This model has the potential to evaluate clinical rehabilitation status of patients with pathological gait, falling, and various foot pathologies when more data of patients with various diseases are accumulated for training.

## Introduction

The human foot constitutes a basic surface that interacts directly with the environment to facilitate bipedal locomotion. It is essential to diagnose foot problems in their early stages for healthy foot management and injury prevention in daily life. Evaluating characteristics of foot pressure distribution is important for typical diagnostic methods [1]. Clinically, foot pressure distribution can be used as a quantitative parameter not only for evaluating anatomical deformity of the foot [2], but also for diagnosing and treating foot functions of patients with chronic plantar fasciitis [3], obesity [4], diabetes [5], and ulcer [6]. It has been reported that reduced medial foot pressure during walking can decrease gait stability, thus increasing the risk of falls in the elderly [7]. Sacco et al. [8] have evaluated foot pressure distribution among patients with diabetic neuropathy during walking. They reported that the pressure was increased throughout the foot as symptoms worsened, with the pressure being centered in the forefoot [8]. Specifically, diabetic neuropathy can lead to slow joint movement and foot deformities, causing alterations in daily movement, including walking. Such movement alteration may cause excessive pressure on specific parts of the foot. Due to the loss of protective sense, the patient might not be able to detect excessive pressure. Repeated load eventually can lead to skin wounds and ulcers [9]. If these changes in foot pressure are detected early in diabetic patients, the development of foot disease and ulcers can be prevented in advance. Therefore, monitoring foot pressure distribution in daily life plays a key role in early diagnosis of disease and tracking of rehabilitation progress [10].

Platform and insole systems are most commonly used to measure foot pressure distribution [11]. Since both systems have their advantages and disadvantages, they are used according to experimental purposes, environmental conditions, and subjects’ conditions [12]. The platform system’s advantages include its solid structure and a large number of sensors arranged in a matrix form, thus allowing for a high resolution. It has been used for analyzing motion such as static posture and walking because it is generally fixed in a flat space and convenient to use [1]. Stewart et al. [13] have analyzed the gait of a patient with gout and asymptomatic hyperuricemia using a MatScan platform device. Their study confirmed that a patient’s pressure distribution was decreased in the hindfoot and hallux, while the pressure distribution was increased in the midfoot [13]. In addition, various studies have quantitatively evaluated treatment and rehabilitation outcomes using a platform system [14, 15]. The platform system’s disadvantages including its high cost and fixation within a space limit its ability to measure a wide range of motion, such as for evaluating daily behavior and walking. Furthermore, there is a problem called “targeting” in which a walking pattern alteration occurs in order to place a foot in the platform [16]. According to previous studies, foot pressure pattern can be altered during walking due to the “targeting” problem [11].

On the other hand, an insole system can overcome some limitations of a platform system. An insole device is a flexible circuit that can be worn in shoes for measurement. It can also be used to measure a wide range of motion without having the “targeting” problem [1]. Pedar-X mobile (Novel Electronics Inc., GmbH, Munich, Germany) and F-Scan systems (Teckscan Inc., Boston, MA, USA) are the most popular insole devices. They have a thin sheet structure in which matrix pressure sensors are arranged [17]. Valentini et al. [18] have analyzed foot pressure during gait in patients with unilateral paralysis and found significant differences between left and right forefoot pressure distributions. The Pedar-X system has also been used to evaluate pressure distribution of gait in the elderly whose hindfoot pressure distributions are lower than those of young adults [7]. However, such systems have a limited on-load time (25 min to 6 h, depending on the system memory) due to data storage and battery power [17]. Its relatively high price also makes it difficult to be used for personal rehabilitation purposes [19]. Specifically, low power consumption, small size and light weight are essential for a mobile health and rehabilitation monitoring system [20]. These problems can be solved by constructing an insole system capable of increasing energy and cost efficiency by attaching less sensors. However, this may affect the ability to obtain distributed pressure information throughout the foot due to limited number of sensors.

Estimating foot pressure distribution with a small number of sensors can be achieved by using an artificial neural network model. As a mathematical model, artificial neural network represents a distributed adaptive computational system utilizing multiple interconnected processing units (neurons), similar to a human neural network. Neurons are distributed in various layers. Signals received from neurons in the previous layer are processed in turn and transmitted to the next layer [21, 22]. Therefore, an artificial neural network model can deduce a successful solution when the input and output have complex and nonlinear relationships. A specialized neural network (deep learning) technique composed of multiple hidden layers is known to have a better performance than traditional neural network structures [23]. When learning a model, deep learning has the advantage of reducing the challenge of extracting inputs by learning main features while passing through the layer step by step, thereby improving the learning accuracy [24]. Sim et al. [25] have used an artificial neural network model to predict ground reaction force data based on insole sensor signals during gait. Their study was done with data from a control group and patients with scoliosis. Estimated correlation coefficients ranged from 0.84 to 0.99. Artificial neural network models including deep learning techniques have also been applied to various studies of an insole system with successful results [26–28].

Identifying and monitoring foot pressure distribution during gait can provide quantitative information for clinical early diagnosis and rehabilitation progress. Currently commercial systems with lots of pressure sensors have limited on-load time and battery power. Thus it is difficult to use them for personal rehabilitation purposes. These problems can be solved by constructing an insole system capable of increasing energy and cost efficiency by attaching less sensors. However, no study has reported the extraction of pressure distribution based on a small number of pressure sensor signals from low-cost insole devices. Therefore, the primary objective of this study was to propose a deep learning model that could predict pressure distribution of the whole foot based on information obtained from a small number of pressure sensors in an insole. The proposed deep learning model was confirmed to have an improved accuracy compared to a traditional machine learning model based on the fuzzy logic. The second objective was to develop a low-cost insole prototype composed of a small number of sensors. The usefulness of the proposed deep learning model for a low-cost insole prototype was also evaluated.

The rest of this paper is organized by first presenting materials and methods consisted of two steps. Firstly, experimental design (participants, apparatus and protocols), model implementation, and model validation are presented to develop a deep learning model. As the second step, application details of the proposed deep learning model using a low-cost insole prototype are presented to confirm the usefulness of the model. Results section then explains overall results including the raw pressure signals and the performances of the models. Discussion section then summarized findings of this study along with study limitations and future research directions. The last section provides conclusions of this study.

## Materials and methods

### Participants, apparatus and experimental protocols

Participants of this study included 20 young adult males (age: 25.3 ± 1.2 years, height: 170.3 ± 4.1 cm, mass: 66.6 ± 6.5 kg) and 20 older adult males (age: 75.5 ± 5.4 years, height: 167.0 ± 3.0 cm, mass: 68.4 ± 8.0 kg) who had no history of musculoskeletal disorders. All experiments were approved by Institutional Review Board of Sungkyunkwan University. Written informed consent was obtained from each participant prior to the experiment.

Walking experiments were performed using a Pedar-X system (Pedar Mobile, Novel Electronics Inc., GmbH, Munich, Germany). Subjects wore insole devices to cover their whole feet. Each subject walked in a straight path of about 10 m, wearing comfortable top and bottom clothes with the Pedar-X system in anti-skid socks. Sufficient pre-gait testing was performed to acquire familiarization with the gait experiment. All participants were instructed to walk at their own comfortable walking speed. Each subject performed five gait experiments. Sensor data were acquired at 100 Hz. Time-series sensor data from the Pedar-X system were normalized to 100% of the stance phase, which was determined as the duration from heel contact to toe off. Extracted gait data were used to construct a deep learning model.

### Long short-term memory (LSTM) deep learning model

**Fig. 1:**
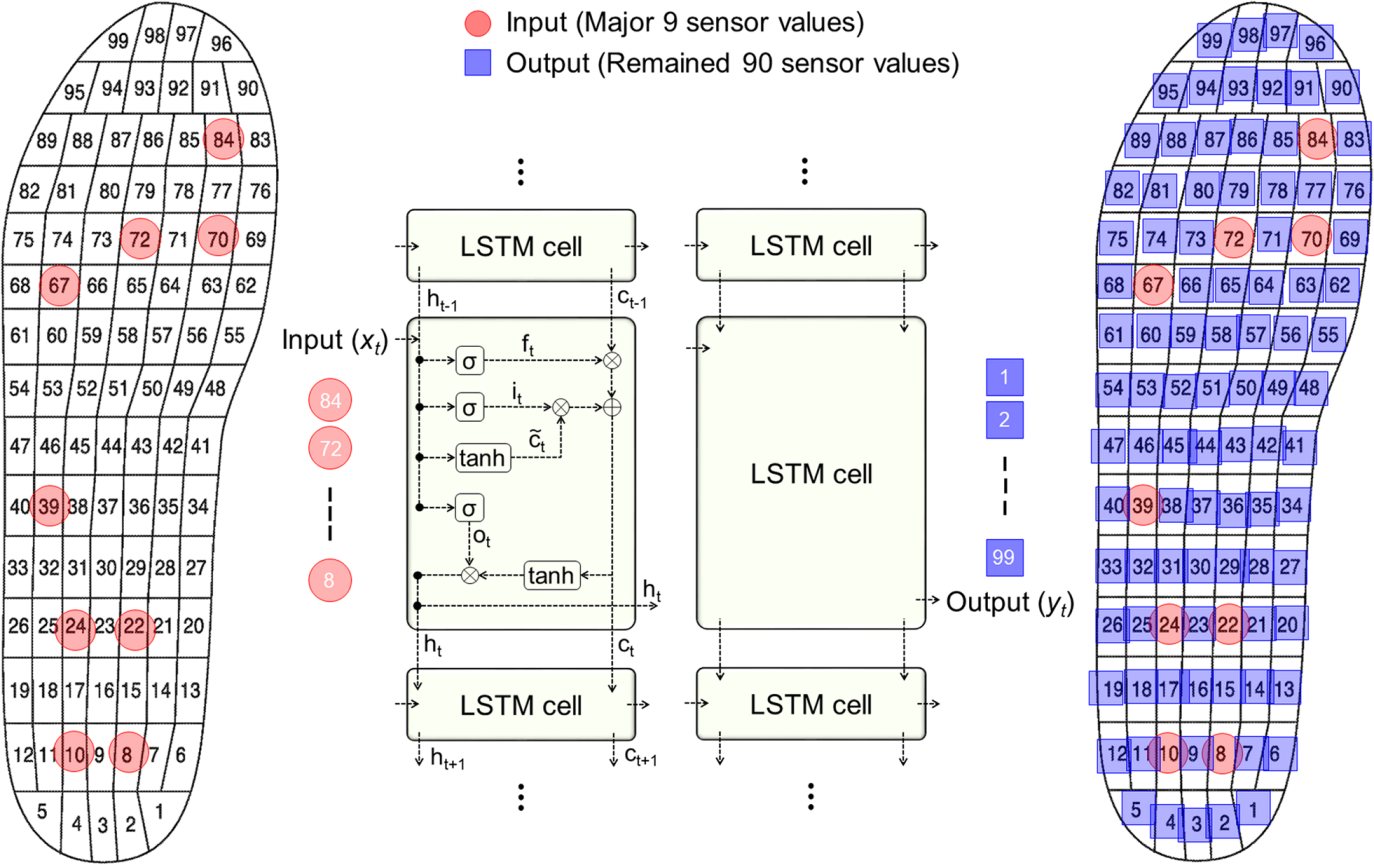
Architecture of the LSTM deep learning model: **x**_**t**_ and **y**_**t**_ represent input and output matrix at each time step, respectively. LSTM model consisted of two hidden layers. Hyperbolic tangent function (**tanh**) was used as the state activation function

Pressure values of nine major sensors (# 8, # 10, # 22, # 24, # 39, # 67, # 70, # 72 and # 84 sensors) and the remaining 90 sensors among a total of 99 sensors in a Pedar-X system were used as input and output for the model, respectively (Fig. 1). The nine major sensor sites anatomically included the heel, metatarsal-phalangeal joints, hallux, and lateral arch. These sites were chosen because pressure is typically concentrated at these sites [29, 30]. The input matrix had a size of 20,000 (number of frames: 20,000 = 40 subjects × 5 trials × 100 frames) × 9 (number of input features). All input values were normalized to be between 0 and 1 in order to increase the efficiency of training and avoid local minima [21].

A LSTM network model was used to predict foot pressure distribution (Fig. 1). The LSTM network model is a type of recurrent neural network. It is characterized by a periodic structure in the form of a directed cycle in hidden layers, making it possible to use an output from the past hidden layer as an input to the current hidden layer. It is a suitable prediction model for continuous time-series signals [31]. In particular, the LSTM network model has a structure in which cell-state is added to retain its long-term dependence on distant past information. In training, the cell-state performs a process of updating or discarding information stored in each memory cell considering the current input and the previous output. This process can reduce errors caused by inputs with little correlation.

The first step in the LSTM network model is to determine what information should be discarded from the cell state. A sigmoid layer consisting of the current input (*x*_*t*_) and the previous output (*h*_*t − 1*_) derives an output value (*f*_*t*_) between 0 and 1. If it is 0, it means to remove it. If it is 1, it means to keep the information. The forget gate is calculated with Eq. 1 as follows:

1 $${\varvec{f}}_{\varvec{t}} = \varvec{\sigma }({\varvec{W}}_{\varvec{f}}\left[{{\varvec{h}}_{\varvec{t}-1}, \varvec{x}}_{\varvec{t}}\right] + {\varvec{b}}_{\varvec{f}})$$

where *W*_*f*_ and *b*_*f*_ denotes weight matrix and bias vector of the forget gate, respectively. The next step is to determine whether new information should be stored in the cell state. The input gate layer plays a role in determining which value (*i*_*t*_) to update as shown in Eq. 2. The tanh layer creates a new candidate value (*c*_*t*_) that can be added to the cell state as shown in Eq. 3:

2 $${\varvec{i}}_{\varvec{t}}=\varvec{\sigma }\left({\varvec{W}}_{\varvec{i}}\left[{{\varvec{h}}_{\varvec{t}-1}, \varvec{x}}_{\varvec{t}}\right] + {\varvec{b}}_{\varvec{i}}\right)$$

3 $${\tilde{\varvec{c}}}_{\varvec{t}}= \mathbf{tanh}\left({\varvec{W}}_{\varvec{c}}\right[{{\varvec{h}}_{\varvec{t}-1}, \varvec{x}}_{\varvec{t}}] + {\varvec{b}}_{\varvec{c}})$$

where *W*_*i*_, *W*_*c*_, *b*_*i*_, and *b*_*c*_ are weight matrix of the input gate, weight matrix of the tanh layer, bias vector of the input gate, and bias vector of the tanh layer, respectively. The new cell state (*c*_*t*_) is then multiplied by the previous cell state (*c*_*t − 1*_) and output value of forget gate (*f*_*t*_), discarding the information determined in the first step. A new cell state is calculated by adding the value obtained by multiplying *i*_*t*_ and *c*_*t*_ as shown in Eq. 4. It creates a new candidate value that can affect the previous cell state (*c*_*t − 1*_).

4 $${\varvec{c}}_{\varvec{t}} = {\varvec{f}}_{\varvec{t}} \times {\varvec{c}}_{\varvec{t}-1} + {\varvec{i}}_{\varvec{t}} \times {\tilde{\varvec{c}}}_{\varvec{t}}\varvec{\sigma }$$

The final step is to determine the output of the repeating LSTM cell. The output gate value (*o*_*t*_) is determined through the sigmoid layer consisting of the current input (*x*_*t*_) and the previous output (*h*_*t − 1*_) as shown in Eq. 5. Additionally, a value between -1 and 1 is extracted from the updated cell state through the tanh function. The output *h*_*t*_ at the current time can be obtained by multiplying the output gate value and tanh*c*_*t*_ as shown in Eq. 6.

5 $${\varvec{o}}_{\varvec{t}} = \varvec{\sigma }({\varvec{W}}_{\varvec{o}}\left[{{\varvec{h}}_{\varvec{t}-1}, \varvec{x}}_{\varvec{t}}\right] + {\varvec{b}}_{\varvec{o}})$$

6 $${\varvec{h}}_{\varvec{t}} = {\varvec{o}}_{\varvec{t}} \times \mathbf{tanh}{\varvec{c}}_{\varvec{t}}$$

Here, *W*_*o*_ and *b*_*o*_ denote weight matrix and bias vector of the forget gate, respectively. In the above process, all weight matrices and bias vectors were initialized to random numbers and updated in the training process of the LSTM network model.

The LSTM model used two hidden layers. A hyperbolic tangent function was used as the state activation function. The model optimizer used an adaptive moment estimation stochastic gradient descent method with a good optimization convergence performance [32]. It was trained in a fully-supervised way with mean-square error set as the loss function. The model was trained for 2,000 iterations with 100 epochs, a mini-batch size of 10, and an initial learning rate of 0.001. Hyper-parameters of the model such as layer numbers were selected to derive the minimum RMSE and avoid over-training based on trial and error [33, 34]. The model was implemented using MATLAB version R2018b (The Mathworks, Inc., Natick, MA, USA) and RTX 2080Ti GPU (4352 CUDA cores, 1665 MHz base clock speed and 11 GB RAM).

The accuracy of the model was evaluated by applying a leave-one-out cross-validation process [35]. Data from 36 subjects were used for training, while data from the four remaining subjects were used for the test. This process was repeated ten times. Pressure values of the 90 remaining sensors were measured with a Pedar-X system and compared with 90 pressure values predicted by the model. These values were compared using root mean square error (RMSE) and relative RMSE (rRMSE, %) values for errors. Correlation coefficients (r) were used to determine the similarity of time-series data patterns.

### Comparing the proposed LSTM model with the adaptive neuro-fuzzy inference system

The performance of the developed LSTM network structure was compared with that of a traditionally used adaptive network-based fuzzy inference system. The adaptive neuro-fuzzy interference system (ANFIS) has a specialized neural network structure combined with fuzzy logic and artificial neural network to determine the optimal membership functions [36]. In general, ANFIS is composed of five layers. The first layer performs fuzzification which transforms input into a range of 0 to 1 using the membership function. The second layer multiplies each neuron’s firing strength of a rule and the third layer normalizes all input signals to allow distinguishment between total firing strength of total rule and firing strength of each rule. The fourth layer performs defuzzification and the last layer summarizes or aggregates all weighted output values [37].

In this study, the neuro-fuzzy structure used a subtractive clustering technique, a technique that combines ANFIS and clustering to estimate each data points into potential cluster center [38]. The number of input membership functions and the type of membership function were set as 2 and *gaussmf*, respectively. The output membership function was set as a linear type. The hybrid optimization technique using least-square and back-propagation at the same time was applied for the training process of the fuzzy inference system. To train the ANFIS model, we specified the maximum epoch to be 50. The ANFIS architecture was constructed with 312 nodes, 150 linear parameters, 420 parameters, and 15 fuzzy rules. The input matrix characteristic for training ANFIS was identical to that of the LSTM model (20,000: number of frames x 9: number of input features). The output matrix size was set as 1 pressure sensor value (20,000: number of frames x 1: pressure value). All pressure values were estimated by repeating the training 90 times. Furthermore, the optimal input membership function (triangular, trapezoidal, gaussian), the number of membership functions (1 ~ 4), and the optimization method (backpropagation, Hybrid) were extracted based on a trial-and-error process to avoid over-training [39]. Similar to the LSTM model, the ANFIS was implemented using MATLAB. The accuracy of the ANFIS was evaluated by applying a leave-one-out cross-validation.

### Application of cost-effective insole system

**Fig. 2:**
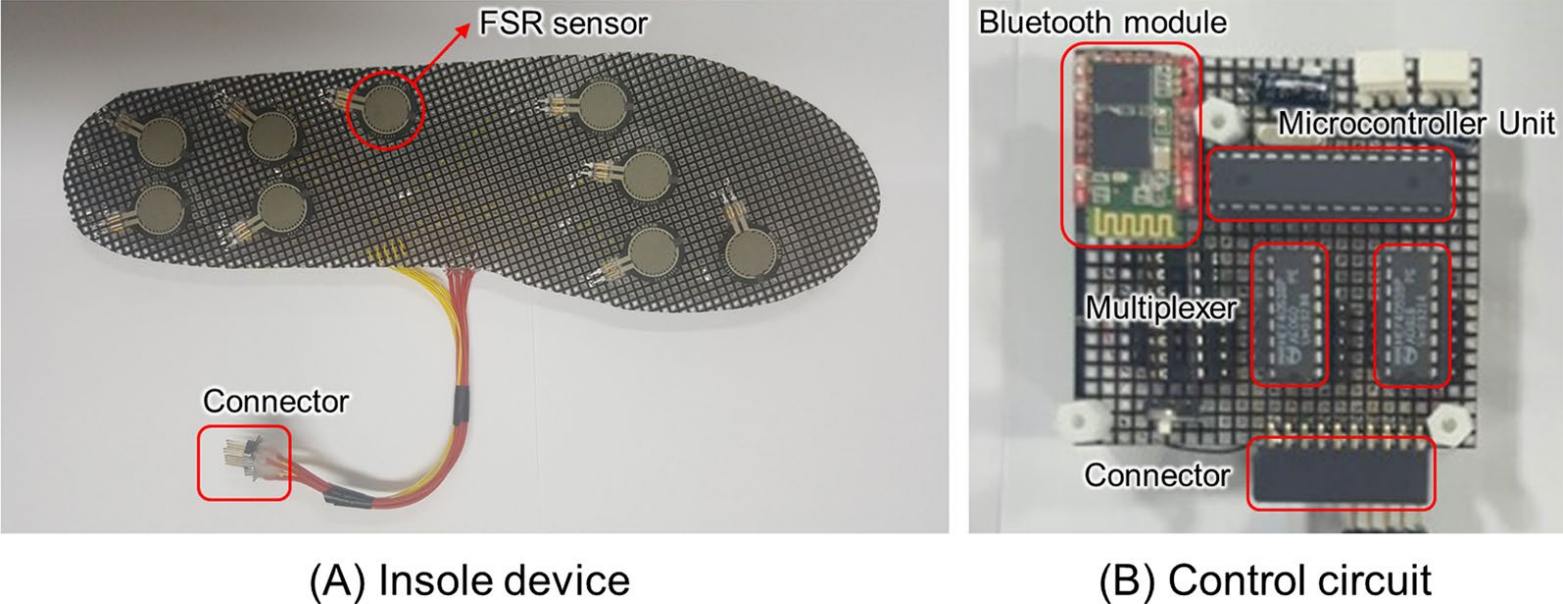
Insole prototype device developed to validate the proposed deep learning models: **A** Insole device, and **B** Control circuit

A low-cost insole system consisting of a small number of pressure sensors was fabricated. Its accuracy was evaluated by applying the proposed LSTM deep learning model. The low-cost insole system consisted of an insole device and a device for data processing and wireless transmission (Fig. 2). Sensors of the insole device were force sensitive resistors (FSRs, FSR 402 Short Tail, Interlink electronics Inc, CA, USA) placed at the same location with nine major sensors in the Pedar-X system as inputs of the LSTM model. They were made in three sizes (250 mm, 260 mm, and 270 mm), considering shoe sizes of subjects. The insole system was made of a flexible substrate to enhance wearability during gait. Its upper part was covered with a thin silicone rubber to protect the sensor and improve the sense of foreign body. The data processing device consisted of a Microcontroller Unit (ATmega 8, atmel), a Multiplexer (HEF4051, Nexperia), a Battery (3.7 V 2000mhA), and a Bluetooth 2.0 V module connected to the insole arch (inner part of the foot) using a ribbon cable. In addition, the processing device was designed to convert voltage output from the nine sensors to digital signals according to changes of load during walking in a structure that could transmit converted digital signals to 100 per second using a Bluetooth module [40]. Raw signals of sensors were extracted from the low-cost insole prototype and acquired with a PC using a LabVIEW software (LabVIEW ver. 20.0, National instruments Corp, Austin, TX, USA).

A gait experiment was additionally performed with five young adult males (age: 24.2 ± 0.8 years; height: 170.1 ± 3.1 cm; mass: 68.7 ± 4.5 kg) and five older adult males (age: 74.1 ± 4.8 years; height: 168.1 ± 4.3 cm; mass: 69.8 ± 4.5 kg), excluding subjects who were used to construct the prediction models. Similar to the gait experiment for model development, the experiment was conducted by wearing comfortable top and bottom clothes. A commercial Pedar-X system was placed parallelly on the top of the insole prototype developed in this study. The system was in anti-skid socks. Both insole systems were fixed through taping to prevent slippage in socks. Sensor data of the developed prototype were acquired at 100 Hz sampling rate. Both insole systems were manually synchronized based on walking events (heel strike and toe-off events). Participants were instructed to walk at their own comfortable walking speed. Each subject performed five gait experiments.

Nine sensors were calibrated to convert sensor signals to force values. Force and voltage data were measured using a digital push-pull gauge meter (DTG-100, DIGITECH Co., Ltd., Japan) equipped with a load cell when the same force was manually applied onto each FSR sensor located on the fixed plate. The relation between these data was then established using an exponential function. The voltage was converted to force value based on the exponential fitting curve, resulting in an average RMSE of 3.7 N and an R-square value of 0.99, respectively. The unit of force was converted from newton [N] into pressure [kPa] divided by the area of the sensor.

The usefulness of the model trained using Pedar-X sensor signals was evaluated by applying the insole prototype fabricated in this study. Pressure values for the 90 remaining sensors measured by the Pedar-X system were compared with 90 pressure values predicted by the LSTM model using input from insole prototype developed in this study (rRMSE and coefficients of correlation). Additionally, foot pressure distributions during stance phase were evaluated for young and older adult groups.

### Statistical analysis

Difference of rRMSE (%) between each model was evaluated using paired t-test. All statistical analyses were performed using PASW Statistics 18 program version 18 (SPSS Inc., Chicago, IL, USA). The significance level was set at *p* < 0.05 and *p* < 0.01.

## Results

**Fig. 3:**
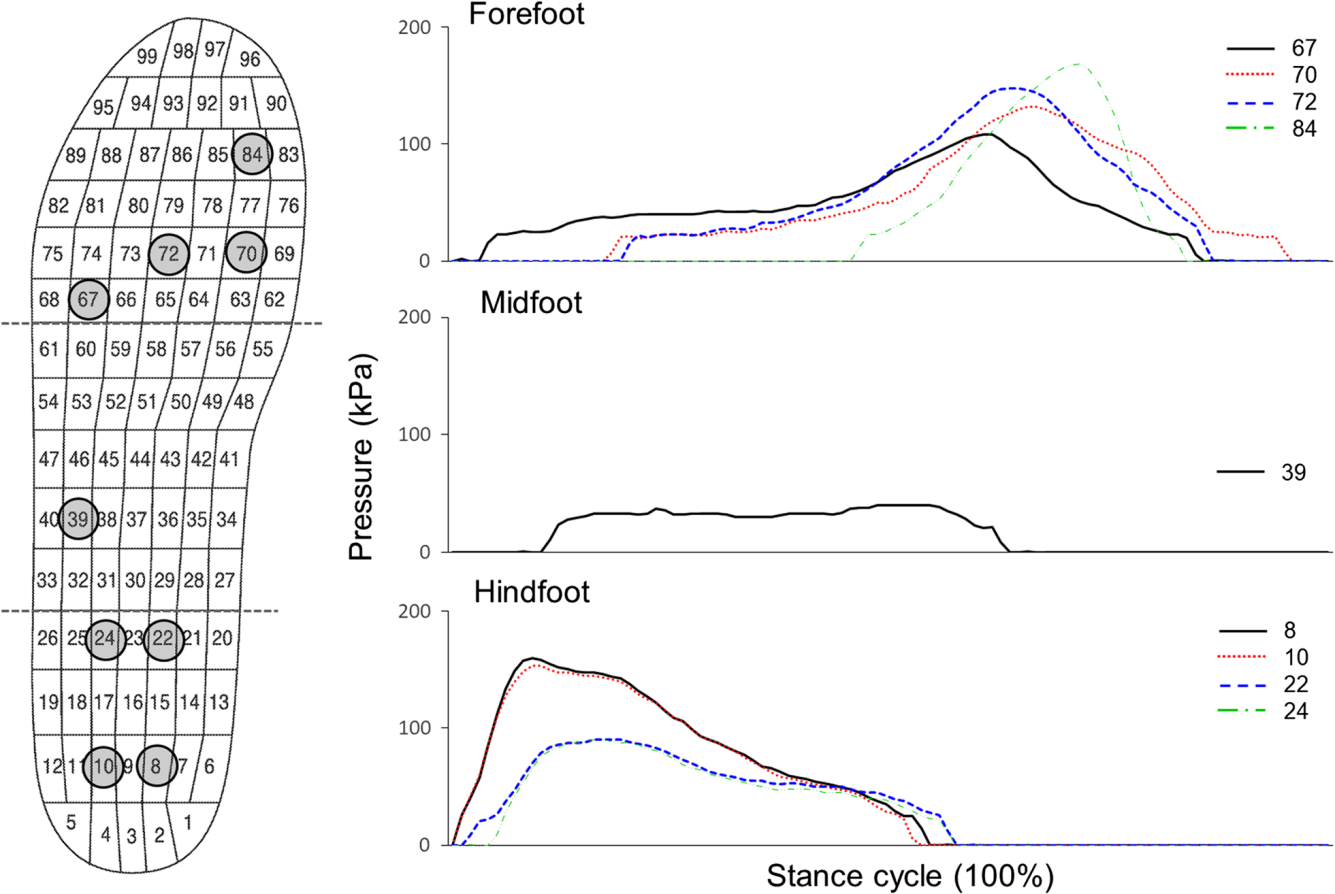
Pressure patterns obtained from nine major sensors during stance phase

Figure 3 demonstrates foot pressure values measured with nine major sensors during the stance phase of a representative subject. The foot was divided into three regions: forefoot, midfoot, and hindfoot. Peak pressure of about 170 kPa was found in the forefoot and the hindfoot. Pressure values for sensors attached to the hindfoot (sensors # 8, 10, 22, and 24) were relatively high in the initial phase, while pressure values for sensors attached to the forefoot (Sensors # 67, 70, 72, and 84) appeared to be high in the end phase. The pressure value for the sensor attached to the midfoot was the lowest (at about 50 kPa).

**Fig. 4:**
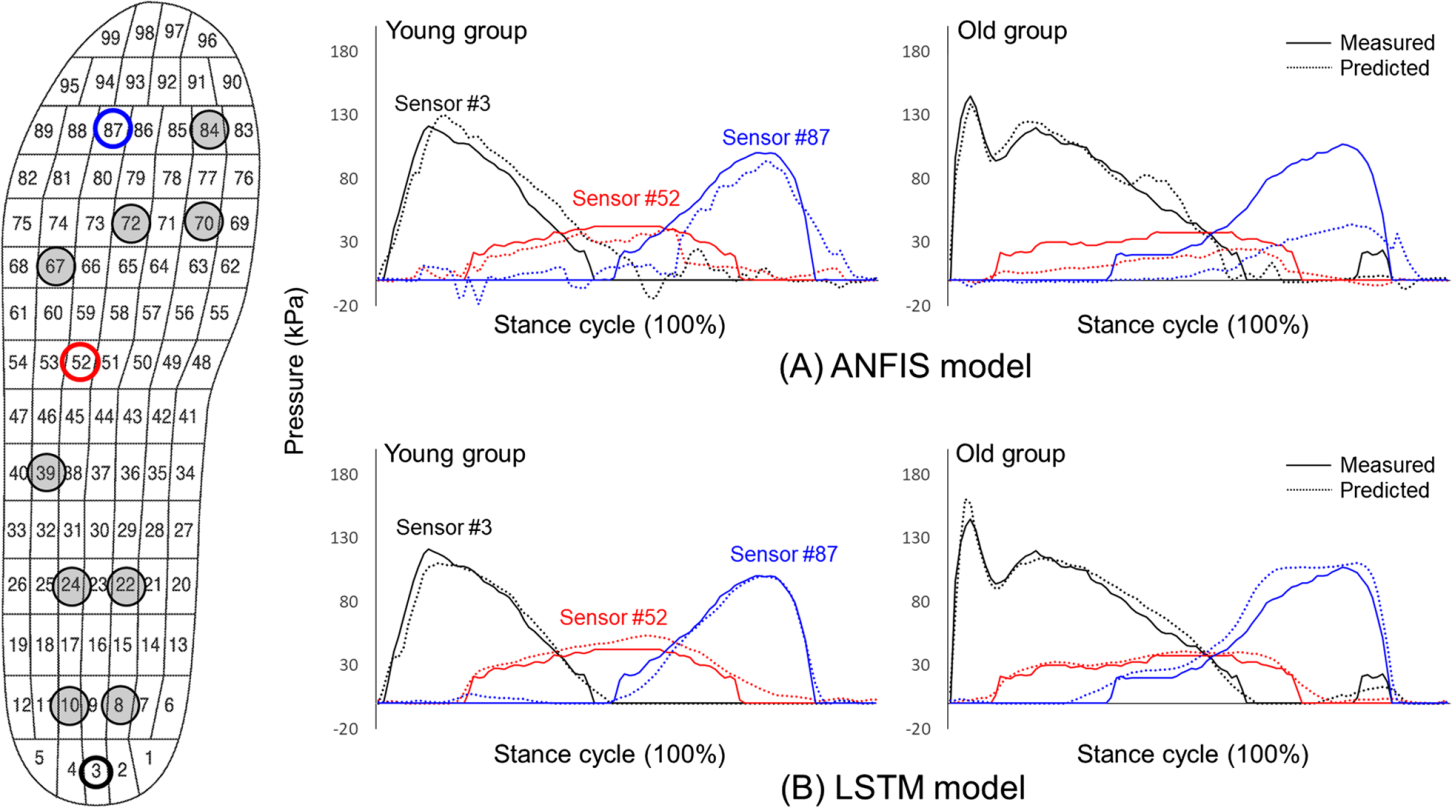
Representative measured (using a Pedar-X system) and predicted (ANFIS and LSTM models) pressure patterns during stance phase

Results of comparison between pressure values predicted by the ANFIS and the LSTM model proposed in this study and measured values using a Pedar-X commercial system are shown in Fig. 4. Representative sensor values (Sensors #3, #52, and #87) by each foot region among 90 predicted sensors were observed. RMSE was 9.4 kPa for forefoot sensors, 6.3 kPa for midfoot sensors, and 5.5 kPa for hindfoot sensors with the ANFIS model. The LSTM network performed better than the ANFIS model, showing RMSEs of 7.0 kPa, 3.7 kPa, and 5.6 kPa for sensors # 87, # 52, and # 3, respectively. The signal pattern with the LSTM model was smoother than that with the ANFIS model.

**Fig. 5:**
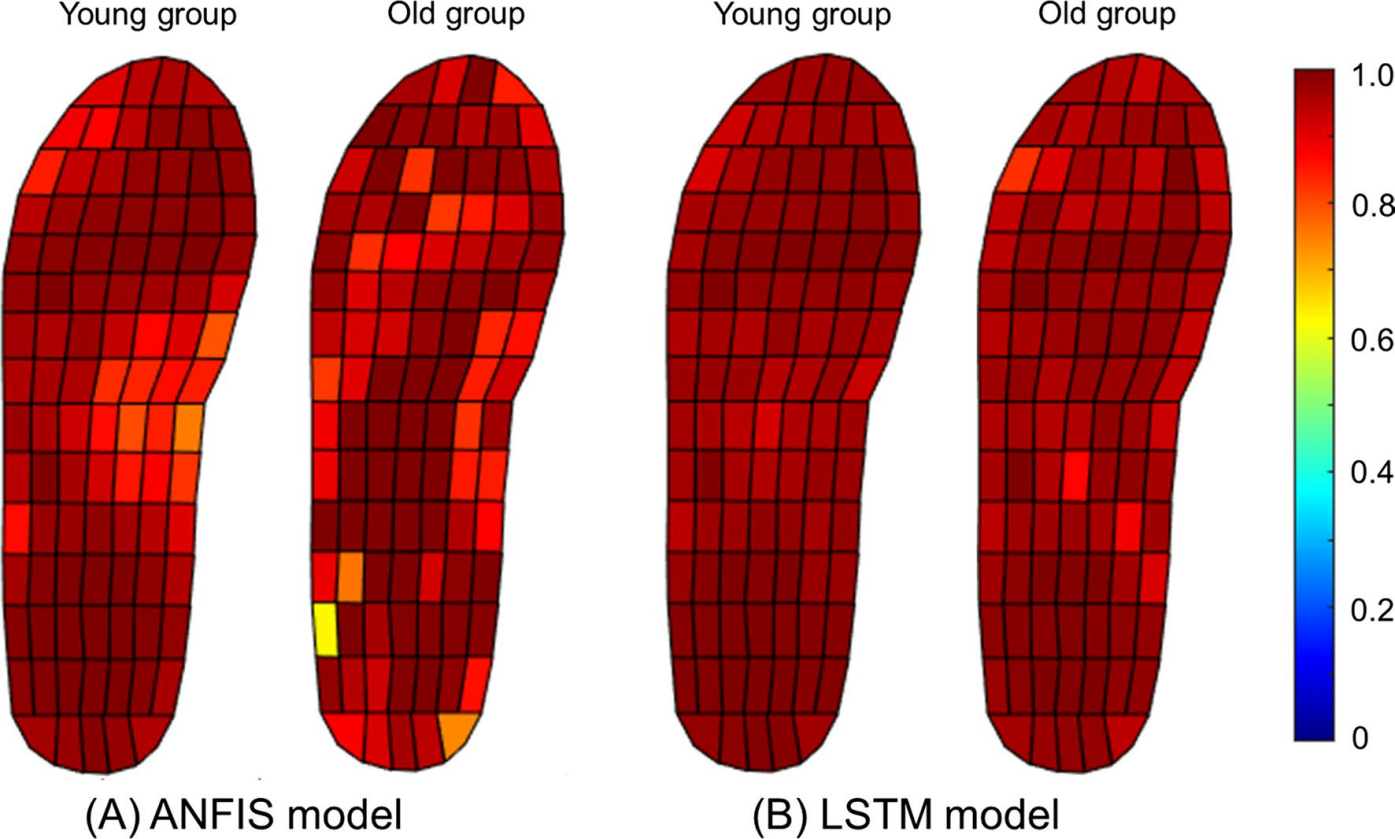
Correlation coefficient between measured (using a Pedar-X system) and predicted (ANFIS and LSTM models) pressure values during stance phase

Figure 5 demonstrates correlation coefficients between measured and predicted values of time series pressure by group and model in a mesh grid using colormap. The average correlation coefficient for the LSTM network model proposed in this study was 0.98 (0.92–0.99), higher than the average correlation coefficient for the ANFIS model, which was 0.94 (0.63–0.99). In addition, the correlation coefficient of the young group (r = 0.99–0.96) was higher than that of the old group (r = 0.99 ~ 0.93) with the LSTM network model.

**Fig. 6:**
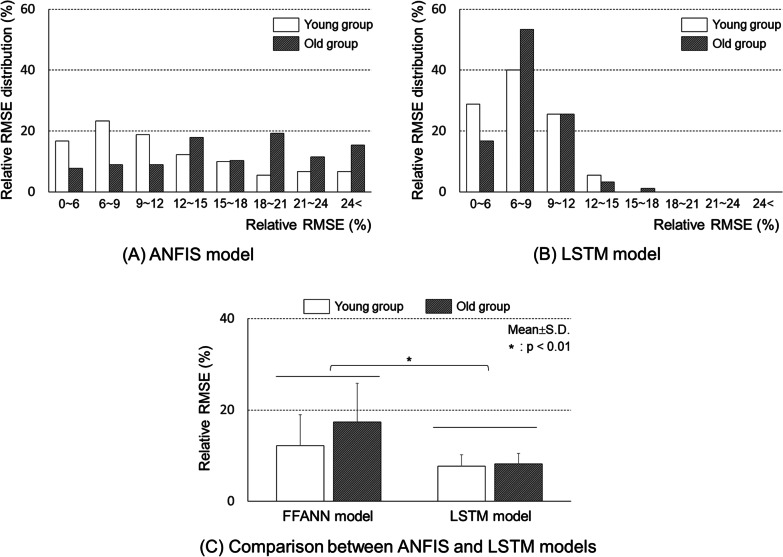
Relative RMSE distribution between measured (using a Pedar-X system) and predicted (ANFIS and LSTM models) pressure values

Results of rRMSE between pressure values measured with the Pedar-X commercial system and those predicted with the model are demonstrated in Fig. 6. In the young adult group, those with rRMSEs of 6-9% accounted for 23%. In the older adult group, those with rRMSEs of 18-21% accounted for 19%, the largest portion in the ANFIS model. In addition, those with rRMSEs greater than 24% were found to account for about 6% in the young adult group and about 15% in the older adult group (Fig. 6A). In contrast, those with rRMSEs of 6-9% had the largest portion (48% in the young adult group and 40% in the older adult group). There was no case where the rRMSE was greater than 18% with the LSTM network model (Fig. 6B). In summary, the average rRMSE of the LSTM network model was 7.9 ± 2.3%, which was significantly lower than that of the ANFIS model (14.5 ± 7.6%) (Fig. 6C). Both prediction models (ANFIS and LSTM network model) had better rRMSE values for the young adult group than for the older adult group. However, rRMSE values were not significantly different between the two groups (*p* > 0.05).

**Fig. 7:**
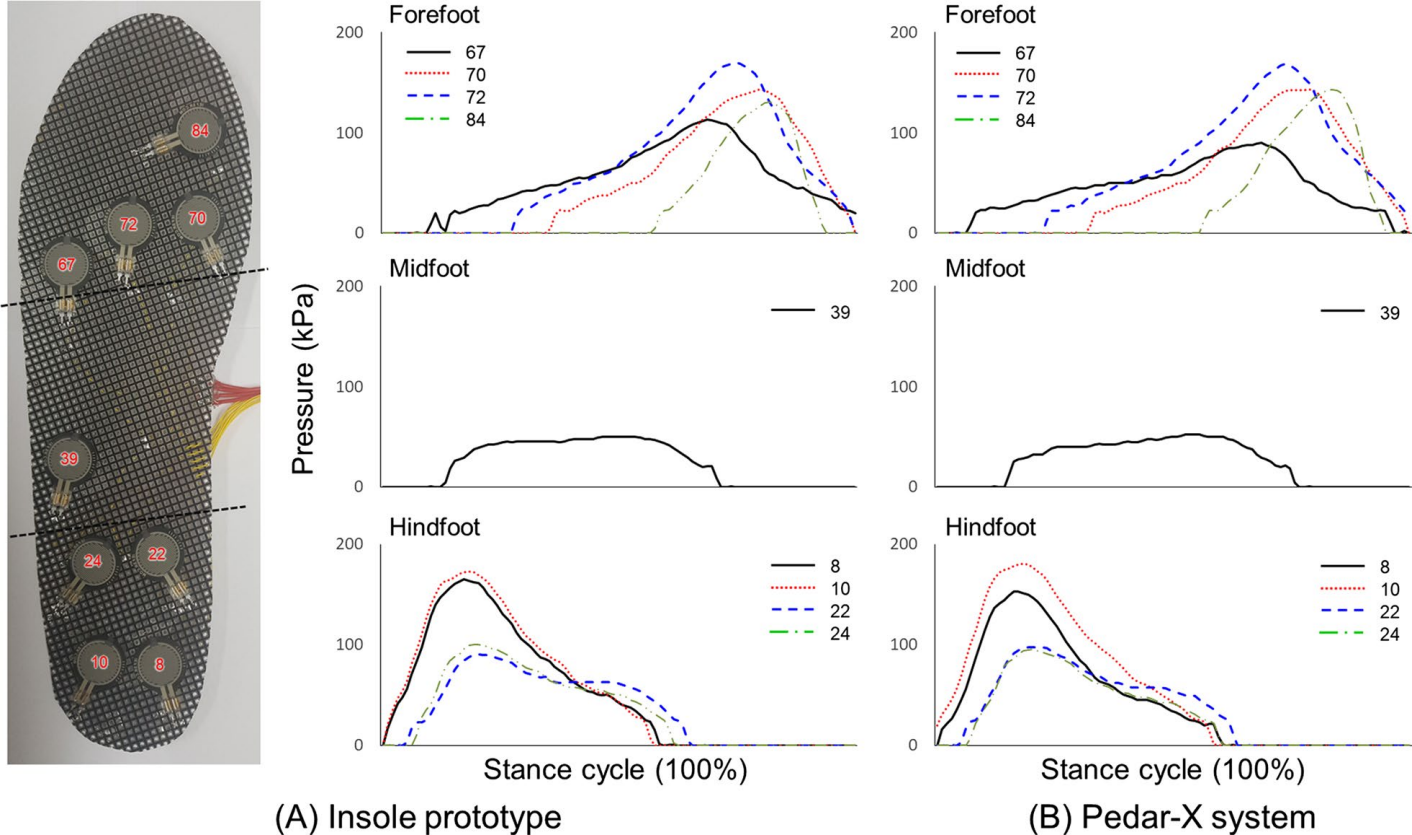
**A** Representative measured pressure patterns using the insole prototype developed in this study and **B** Corresponding pressure patterns using a Pedar-X system during stance phase

To examine the applicability of the proposed LSTM network model, pressure values of the nine FSR sensors during stance phase were acquired from a self-manufactured insole prototype (Fig. 7A). Additionally, representative pressure values of the nine major sensors in the Pedar-X commercial system during stance phase are demonstrated in Fig. 7B to compare the similarity of pressure values extracted with the insole prototype. Pressure values measured using sensors of the insole prototype during the stance phase appeared to be similar to pressure values measured using the Pedar-X system, with an average RMSE of 6.9 ± 1.7 kPa and r of 0.98 (0.90–0.93).

**Fig. 8:**
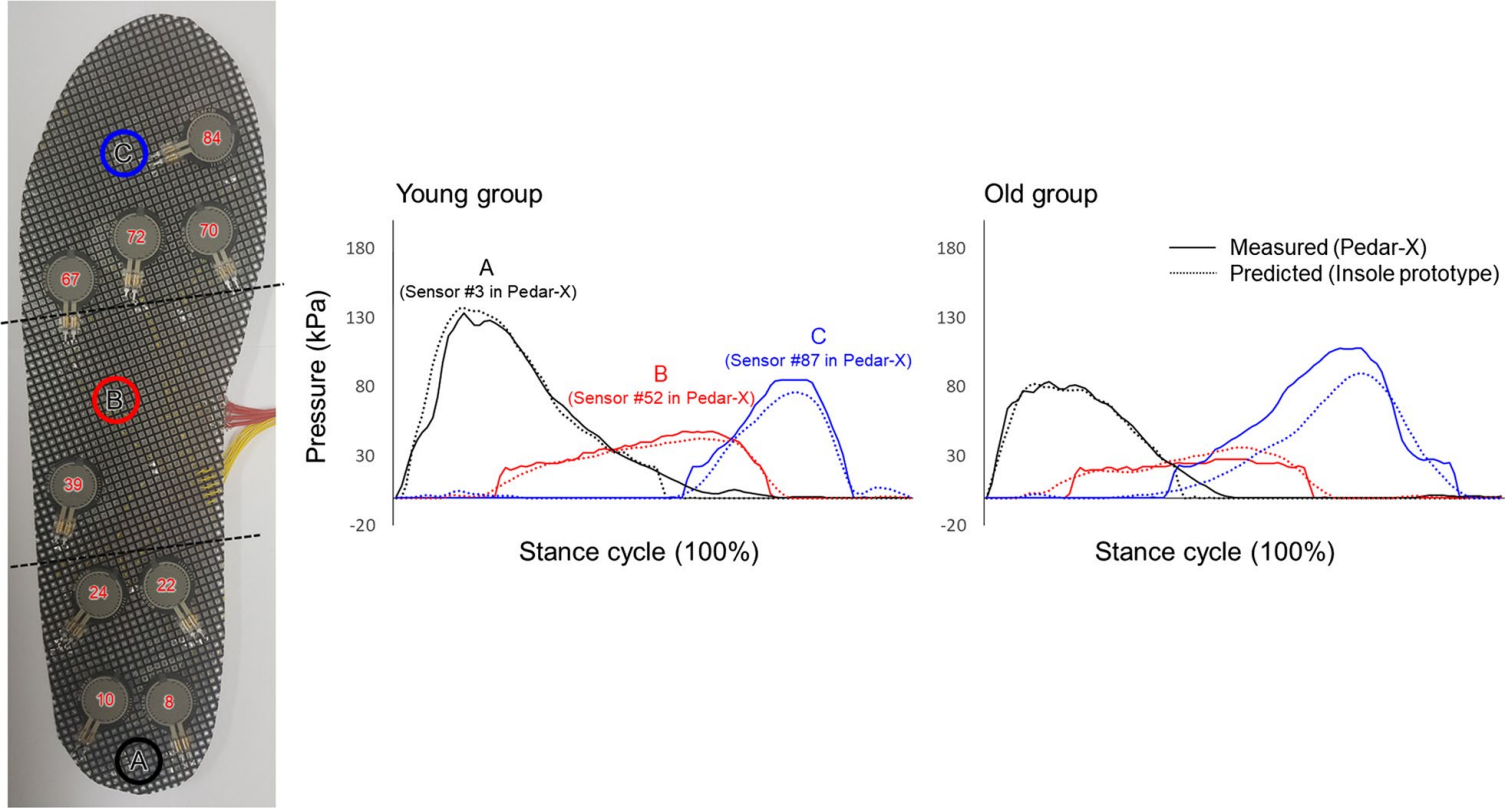
Representative measured (using a Pedar-X system) and predicted (using the proposed LSTM model from nine FSR sensor signals of the insole prototype as model input) pressure patterns during stance phase

Figure 8 demonstrates results of comparison between pressure values measured using a commercial Pedar-X system and those predicted by the LSTM model proposed in this study. Pressure values of representative sensors located to ‘A’, ‘B’ and ‘C’ in insole prototype (the same location with sensors #3, #52, and #87 in the Pedar-X system) by each foot region among 90 predicted sensors were observed. RMSE was 10.8 kPa for forefoot sensors, 3.8 kPa for midfoot sensors, and 6.0 kPa for hindfoot sensors with the LSTM model. The model had more similar plot pattern and better RMSE values for the young adult group than for the older adult group.

**Fig. 9:**
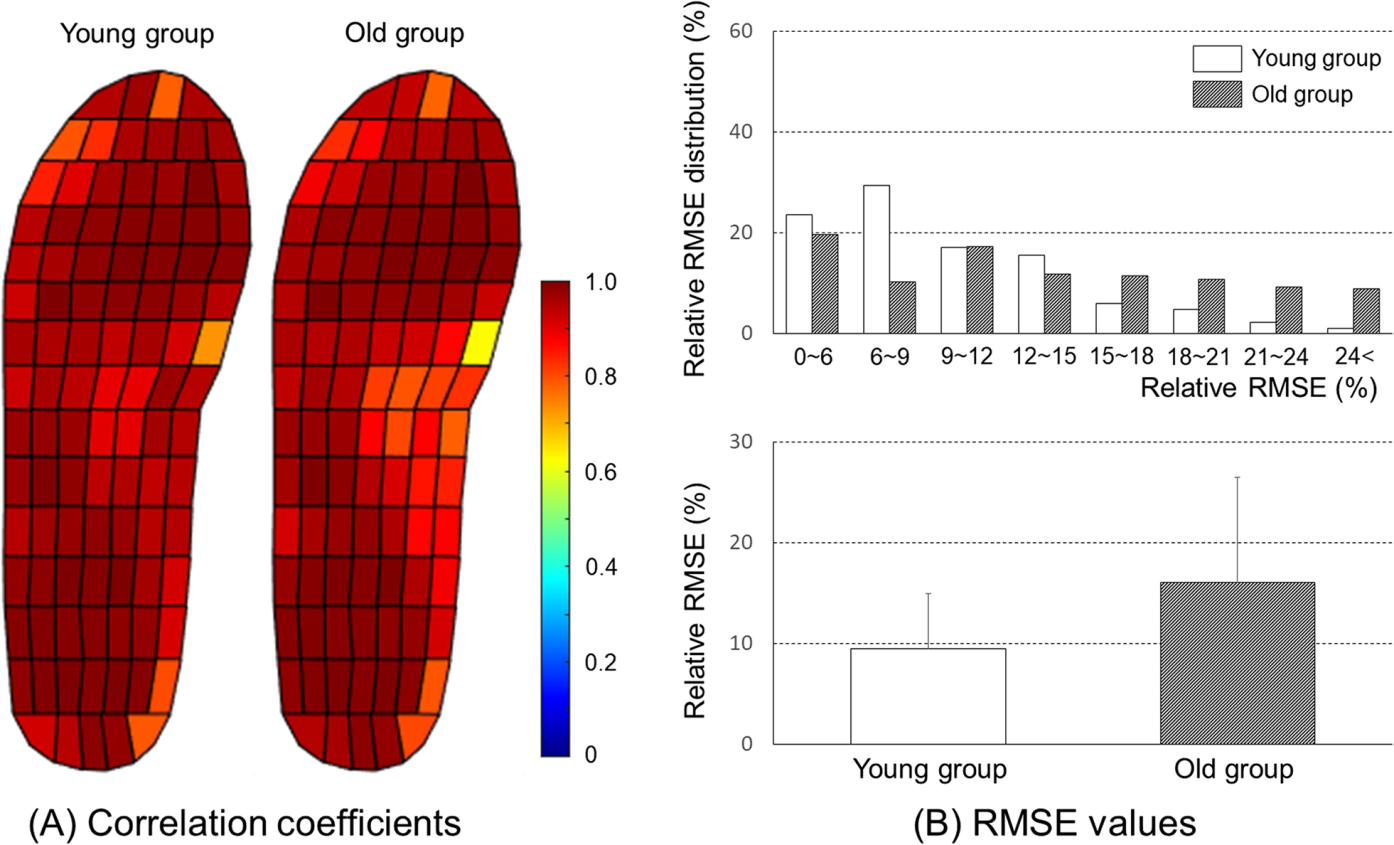
**A** Correlation coefficients and **B** Relative RMSE distribution between measured (using a Pedar-X system) and predicted (using insole prototype and LSTM model proposed in this study) pressure patterns

Correlation coefficients and rRMSEs between pressure values measured with the Pedar-X system and predicted with the LSTM model using input of insole prototype are demonstrated in Fig. 9. The correlation coefficient was 0.73 to 0.97 for the young adult group and 0.63 to 0.96 for the older adult group, showing excellent predictive performance. In the young adult group, those with rRMSE less than 12% accounted for 70%. However, those with rRMSE less than 12% accounted for about 47% in the older adult group. The average rRMSE of the proposed LSTM model was 12.7 ± 7.4%. The proposed LSTM model had better rRMSE values for the young adult group (9.5 ± 5.5%) than for the older adult group (16.0 ± 10.4%).

## Discussion

Monitoring of foot pressure distribution in daily life plays a key role in early diagnosis of disease and tracking of rehabilitation progress. A cost-effective insole system with a few pressure sensors allows individuals to use it outside the traditional clinic and clinicians for remote supervision. This study investigated a novel deep learning model that could estimate pressure distribution of the whole foot region using pressure values of nine major sensors in foot during the stance phase. The proposed LSTM deep learning model had an improved accuracy compared to the traditional ANFIS. Additionally, an insole prototype with nine FSR sensors was fabricated and the usefulness of the proposed LSTM deep learning model was evaluated using nine FSR pressure values of the insole prototype as model input.

A common method to evaluate foot pressure during walking is to use masking, which divides the sole into several regions of interests and analyzes them to provide clinically important information [41]. In previous studies, these masks were used based on various numbers and regions. Seven plantar regions (heel, midfoot, first metatarsal, second metatarsal, metatarsal three to five, the hallux and the lesser toes) were divided to mask the foot to evaluate foot pressure distribution during walking in patients with gout and asymptomatic hyperuricemia [13]. Hessert et al. [7] have used nine anatomical masks including medial and lateral calcaneus, medal and lateral arch, first metatarsal, metatarsals two and three, metatarsals four and five, hallux, and toes. Areas commonly suggested in various studies are the heel, lateral midfoot, metatarsal and toes, which are known to play a role in supporting the most body weight and controlling the balance of the human body during walking and various movements [42]. In this study, these commonly suggested plantar areas were subdivided into nine subregions: heel (4 regions), midfoot, metatarsal (3 regions), and hallux [43]. Specifically, nine locations showing the largest pressure values in each region were selected based on a previous study using footprints [30]. Pressure values ​​extracted from these nine locations were regarded as main features of machine learning for estimating foot pressure distribution.

The LSTM model for estimating 90 pressure distributions using the selected nine main sensor pressure signals showed a rRMSE of about 8% (Fig. 6 C). In machine learning models, the black box structure of inputs and outputs makes it very difficult to interpret physical insights and obtain direct evidence to determine the accuracy of pressure distribution estimates for the stance phase [33]. However, the pressure distribution can be estimated because pressure values ​​of these main nine sensors are known to be concentrated during walking. In addition, pressure values ​​of neighboring sensors are highly correlated with them of each main sensor. In various previous studies analyzing the pressure distribution, a continuous and smooth change in the pressure value has been observed along the periphery of the area where the concentrated pressure value appears [7, 13]. In general, it is known that the higher the dependency between input and output in a machine learning model, the better the model performance [44]. Therefore, the training performance of the model can be secured based on the dependence of the area where the peak pressure value appears and the surrounding locations. However, it is difficult to guarantee that locations of the nine sensors determined in this study are absolute positions for accurately estimating foot pressure distribution. Therefore, it is necessary that further consideration is required for optimal sensor locations to predict foot pressure distribution.

This study was conducted to determine if LSTM deep learning model could predict pressure distribution of the whole foot during gait. The usefulness of this model was also determined by applying it to a low-cost insole device. Although it is difficult to conduct direct comparisons since there is no similar previous study, Ardestani et al. [45] have proposed an artificial neural network model to predict lower limb joint torque during gait using EMG signals from eight lower limb regions and ground reaction force information as input. The correlation coefficient was 0.94 or higher with a relative error of 10% or less. In a study that predicted 3-axis ground reaction force information using insoles during gait, the correlation coefficient was between 0.836 and 0.993 and the relative error was about 8–18% [25]. Liu et al. [46] have reported that an artificial neural network model for predicting joint torque has an excellent performance if the correlation coefficient is 0.9 or higher and if the relative RMSE is within 15%. The correlation coefficient of the model developed in the present study was 0.92 or higher, with a rRMSE of less than 8%. Even the validation using a low-cost insole prototype system showed a correlation coefficient of 0.9 for the LSTM network model. Since this model had low rRMSE values and high correlation coefficients in training and test processes, it could be concluded that this model was efficiently constructed for predicting foot pressure distribution.

In this study, we proposed a model for predicting pressure distribution using LSTM deep learning model. It could effectively estimate time series data. Its performance was evaluated by comparing the model with a traditional fuzzy logic model. The LSTM network model is believed to be able to construct a more accurate model than the traditional fuzzy logic model because data at the previous point in time are entered as input of the LSTM network model that can affect the output. LSTM as a type of circulatory neural network is composed of a complex network that uses both output of time *t-1* and input of time *t* to output results of time t in time series information [16]. Therefore, time series-based output information can be estimated continuously at each point. This study also produced smooth results compared to those using a fuzzy logic machine learning model (Fig. 4).

The performance of the proposed model for the young adult group was generally higher than that for the older adult group, although in-depth statistical analysis was not performed (Figs. 6 and 9). It is difficult to identify the exact cause of the performance difference due to the characteristics of the machine learning model. However, the difference in variability of the raw input data (pressure values of nine major sensors) could be one of factors contributing to the performance difference between young and older adult groups. The range of age of older adult group participated in this study varied from 69 to 80 years old. Accordingly, the deviation of walking characteristics of the older adult group might be higher than that of the young adult group. From the training model perspectives, it can be estimated that the increase of data deviation can act as a bias, leading to a lower performance for the older adult group than for the young adult group. As a future study, more robust training models by using gait big-data of various age groups and including age variable as one of the inputs are needed.

For robust validity of developed models, an insole prototype was fabricated using commercial low-cost FSR sensors. In general, validation results of the low-cost FSR sensor showed lower correlation coefficients than cross-validation results due to different sources of input values. An error might have occurred in the process of sensor calibration when converting voltage signals to pressure values. Even if accurate sensor calibration was performed, the combination of sensor values in the stance phase might be different from characteristics of the training data of the model. However, in this study, the validity of the model was verified using sensors with completely different sources of input to demonstrate the usefulness of the model. Results revealed that the LSTM deep learning model showed a high accuracy with a correlation coefficient of 0.9. Since no such utility validation associated with machine learning in the field of biomechanics has been done, the presented validation process and results are considered to be unprecedented, original data.

This study has a few limitations. First, data of patients with various diseases were not used for the training of the model. Since the machine learning model was constructed based on training data, results of the current model might not be representative if data from patients with various diseases (cerebral palsy, pressure sore, etc.) are used as inputs. However, this study aimed to present a new methodology to predict foot pressure distribution. Further studies are needed to expand the model by including data from patients with various diseases. Second, various artificial intelligence techniques were not used in this study. In addition to artificial neural networks, techniques such as SVM, random forest, and decision tree algorithm have been used in other studies. Therefore, it is necessary to comparatively evaluate the accuracy, stability, and performance of different algorithms using various techniques.

## Conclusions

The present study proposed a LSTM deep learning model that could estimate pressure distribution of the whole foot region using main pressure sensor values of the foot in the stance phase movement. Cross-validation results showed a high accuracy, with correlation coefficients of 0.92 to 0.99 and rRMSE values of about 8.0%. In addition, the developed LSTM model was applied by fabricating a low-cost insole prototype with a small number of pressure sensors. Correlation coefficients measured with a commercial insole system were compared with those predicted with the model. The usefulness of the model was confirmed, showing correlation coefficients of 0.63 to 0.97. This is the first study to predict foot pressure distribution using input data of a small number of pressure sensors. This model can be used as an algorithm to develop a portable smart insole system to monitor age-related physiological and anatomical alterations in foot. In addition, this model has the potential to evaluate clinical rehabilitation status of patients with pathological gait, falling, and various foot pathologies by further improving data training databases of patients with various diseases.

## Data Availability

Data and materials are available from the authors upon reasonable request.

## Abbreviations

LSTM: Long short-term memory
ANFIS: Adaptive neuro fuzzy inference system
COP: Center of pressure
RMSE: Root mean square error
rRMSE: Relative root mean square error
FSR: Force sensitive resistors
